# The influence of executive functions on eye-hand span and piano performance during sight-reading

**DOI:** 10.1371/journal.pone.0285043

**Published:** 2023-05-02

**Authors:** Kyoko Imai-Matsumura, Megumi Mutou

**Affiliations:** 1 Graduate School of Education, Bukkyo University, Kyoto, Japan; 2 Kobe City Board of Education, Kobe, Hyogo, Japan; University Ceuma, BRAZIL

## Abstract

The ability to perform an unrehearsed piece of music, or sight-read, is a skill required by music performers. In sight-reading, the performer reads and plays the music simultaneously, which requires the coordination of visual, auditory, and motor processing. While performing, they display a characteristic called eye-hand span, in which the part of the score being looked at precedes the part being played. They must recognize, decipher, and process the score in the time between reading a note and playing it. An individual’s executive function (EF) that control their cognition, emotions, and behavior may be involved in overseeing these individual movements. However, no study has investigated how EF is related to the eye-hand span and performance during sight-reading. Therefore, the purpose of this study is to clarify the relationships among EF, eye-hand span, and piano performance. Thirty-nine Japanese pianists and college students aspiring to be pianists with an average of 33.3 years of experience participated in this study. They performed sight-reading of two types of music scores with different difficulty levels while their eye movements were measured using an eye tracker to obtain their eye-hand span. The core EFs of inhibition, working memory, and shifting, were measured directly for each participant. Piano performance was evaluated by two pianists who did not participate in the study. Structural equation modeling was used to analyze the results. The results showed that auditory working memory predicted eye-hand span (*β* = .73, *p* < .001 in easy score; *β* = .65, *p* < .001 in difficult score), and eye-hand span predicted performance (*β* = .57, *p* < .001 in easy score; *β* = .56, *p* < .001 in difficult score). Auditory working memory did not directly affect performance, but through eye-hand span. The eye-hand span for easy scores was significantly greater than that for difficult scores. Furthermore, in a difficult music score, the shifting ability predicted higher piano performance. These suggest that the input of notes from the eyes becomes sound in the brain and activates the auditory working memory, which is then transmitted to finger movement, resulting in piano performance. In addition, it was suggested that shifting ability is also needed to perform difficult scores.

## Introduction

Musicians need to be able to sight-read, which is the ability to play a music score for the first time without preparation [1, 2]. Sight-reading occurs not only in situations where a player is performing a novel piece of music, but also in situations where he or she is asked to provide accompaniment. During sight-reading, which is performed while looking at the music score, a performer’s eye movements on a score are characterized by an "eye-hand span" (EHS) in which the part of the score being looked at precedes the part being played [3, 4]. In other words, the performer’s gaze is always positioned in front of the note produced by the instrument, resulting in a span between the note being looked at and the note played by the hand.

During sight-reading, the score must be simultaneously read and converted into a series of motor responses, which requires the coordination of visual, auditory, and motor processing [5]. This allows the musician to recognize, decode, and process the notes that are about to be played, as well as the possible difficulties in playing them, and to plan gestures accordingly [6]. This coordination is essential to ensure adequate perceptual and memory resources between reading and playing each note to prepare motor responses to musical notation [7]. Kinsler and Carpenter [7] proposed a model of eye movements during performance in Sight-reading. The model shows the flow of visual information of individual notes or groups of small notes encoded and processed in the brain, once placed in a buffer and then played. They state that the capacity of the buffer determines how far ahead one can read during the performance. The buffer they are describing can be thought of as working memory (WM). Furneaux and Land [8] also stated that information processed in this way must be stored until the performance reaches the appropriate part of the sequence. Furthermore, a series of actions such as reading music, fingering the keyboard according to the score, and listening to the sound played need to be ordered and controlled. In this context, an executive function (EF) that oversees the control of inhibition and switching, as well as WM, is considered necessary [9, 10].

The EF is the ability to control cognition, emotion, and behavior [11] and refers to a family of top-down mental processes [12, 13]. It is generally agreed that there are three core EFs: inhibition, WM, and cognitive flexibility [10, 14]. One of the core EFs, inhibitory control, refers to the ability to control one’s attention, behavior, thoughts, and emotions in order to override strong internal tendencies or external temptations and do what is most appropriate or necessary [9, 10, 14]. The second, WM is the ability to temporarily store information, process and use the stored information [9]. WM is classified into visuospatial WM, which processes visual information, and auditory WM, which processes auditory information. The third core EF is cognitive flexibility, which is an individual’s ability to shift their perspective spatially, interpersonally, and thoughtfully. These three core EFs control cognition, emotion, and behavior [10, 14].

There have been several reports on the EF of pianists. For example, in a study by Kopiez and Lee [15, 16], a significant positive correlation between sight-reading task performance and WM was shown in pianists with a wide range of sight-reading skills. Further, Meinz and Hambrick [17] examined the extent to which deliberate practice (i.e., engagement in activities specifically designed to improve performance) and WM predicted performance on a sight-reading performance task. The results showed that both were predictive of sight-reading performance, with deliberate practice predicting 45.1% and WM predicting 7.4% of the performance. These results indicate that although deliberate practice is important, WM is also essential to sight-reading. Furthermore, this study also reported that years of piano training did not correlate with WM capacity. This finding is supported by another study that compared the WM of musicians with more than 6 years of formal instrumental music training from before the age of 10 with that of non-musicians with no musical experience [18].

Although there have been some studies on the EF of performers, there is a paucity of studies reporting on the relationship between EHS and EF. One of the reasons for this is the difficulty of measuring EHS. EHS has previously been measured in a variety of ways, about half of which have been conducted in the last decade based on advances in eye-tracking technology. Studies conducted prior to these advancements were not able to accurately measure EHS [6]; therefore, there are few prior studies that have provided accurate information.

The two methods for measuring EHS that have been previously used include: 1) measures of distance, which make it possible to evaluate the quantity of information manipulated by musicians between the moment when they visually fixate on a note and the moment they play it (these measures are obtained by measuring the distance between the fixation point and the virtual position of the hand on the score); and 2) latency measures, which are used to evaluate the amount of time for which the information is maintained in WM [6]. On the other hand, Huovinen et al [19] performed EHS in the reading direction, and at any given time, they took the music note played and measured how far the eyes were from it. They then named it the "forward projection method".

Additionally, EHS is measured using an eye tracker. The number of notes [20, 21] and the number of beats [2, 20–22] up to the note on which the gaze stops at that moment are measured with respect to the note played. Another method measures the latency from the time of one’s gaze fixating on a note to the time of its performance. This measure is expressed in milliseconds (ms) [2, 19, 21, 23, 24].

Factors known to influence EHS include a musician’s level of expertise [4, 8, 25] and the complexity of the score [6]. Skilled musicians have been shown to exhibit greater EHS (distance) than unskilled individuals [8, 25, 26], despite differences in the way experts are classified [6]. Furthermore, EHS is also affected by the complexity of the score, depending on the pianist’s performance skill [19]. The EHS of the best performers increased when the score was easy and decreased when it was complex, but this pattern was not found in performers with low performance ability [19].

However, no studies have measured each element of EHS and EF, and examined the relationships between them. Based on the literature review above, EHS can best be measured through the use of eye-tracking on skilled players during a sight-reading task. In addition, since EHS is affected by the difficulty of the score [19] and the proficiency of the pianist [15, 16], it is important to examine and compare the pianist’s performance ratings using scores of different difficulty levels.

## The present study

In this study, we focused on the following research issues:

1. In a group of skilled piano players, to what extent does each subcomponent of their EF predict EHS during sight-reading, as measured by via eye-tracking? We predicted that WM would most strongly predict EHS among the subcomponents of EF.
2. Does EHS prediction differ for two scores of different complexities? Based on the results of previous studies, we predicted that EHS would be larger for the less complicated and easier scores in comparison to the complex and difficult scores. Thus, we predicted that the relationship between EF and EHS would depend on the complexity of the score.
3. Do EF and EHS during sight-reading predict performance ratings? We predicted that sight-reading performance would be affected by EHS and each subject’s EF.

## Materials and methods

### Participants

The participants were Japanese pianists and college students aspiring to be pianists. Candidates for participation were informed in writing and verbally about the purpose of this study, details of what is involved in participation, and ethical considerations. We explained to the candidates in writing and verbally that they had the right to refuse to participate in the study, that participation was voluntary, and that non-participation would not have any negative consequences. Forty-one individuals agreed to participate in writing. Though the participants included two 19-year-old college students, we did not request consent from their parents, as we did with the other participants. The present research was approved by the Bukkyo University Human Research Ethics Review Committee (No. 2021-4-A) and conducted in accordance with the Declaration of Helsinki.

Thirty-nine participants (8 men and 31 women) were included in the final sample after excluding two participants whose EHS could not be measured. The participants’ ages ranged from 19 to 65 years (*M* = 36.5 years, *SD* = 15.4), and their piano playing experience ranged from 14 to 62 years (*M* = 33.3 years, *SD* = 15.5). Authors did not have access to information that could identify individual participants after data collection.

### Music scores

Scores were selected from the Sight Playing Workbook in the YAMAHA Music Ability Test System. This system ranges from grade 13 (the easiest) to grade 2 (the most difficult), and is used in more than 30 countries. We received permission from YAMAHA for the use and publication of these music scores in this study.

**Fig 1:**
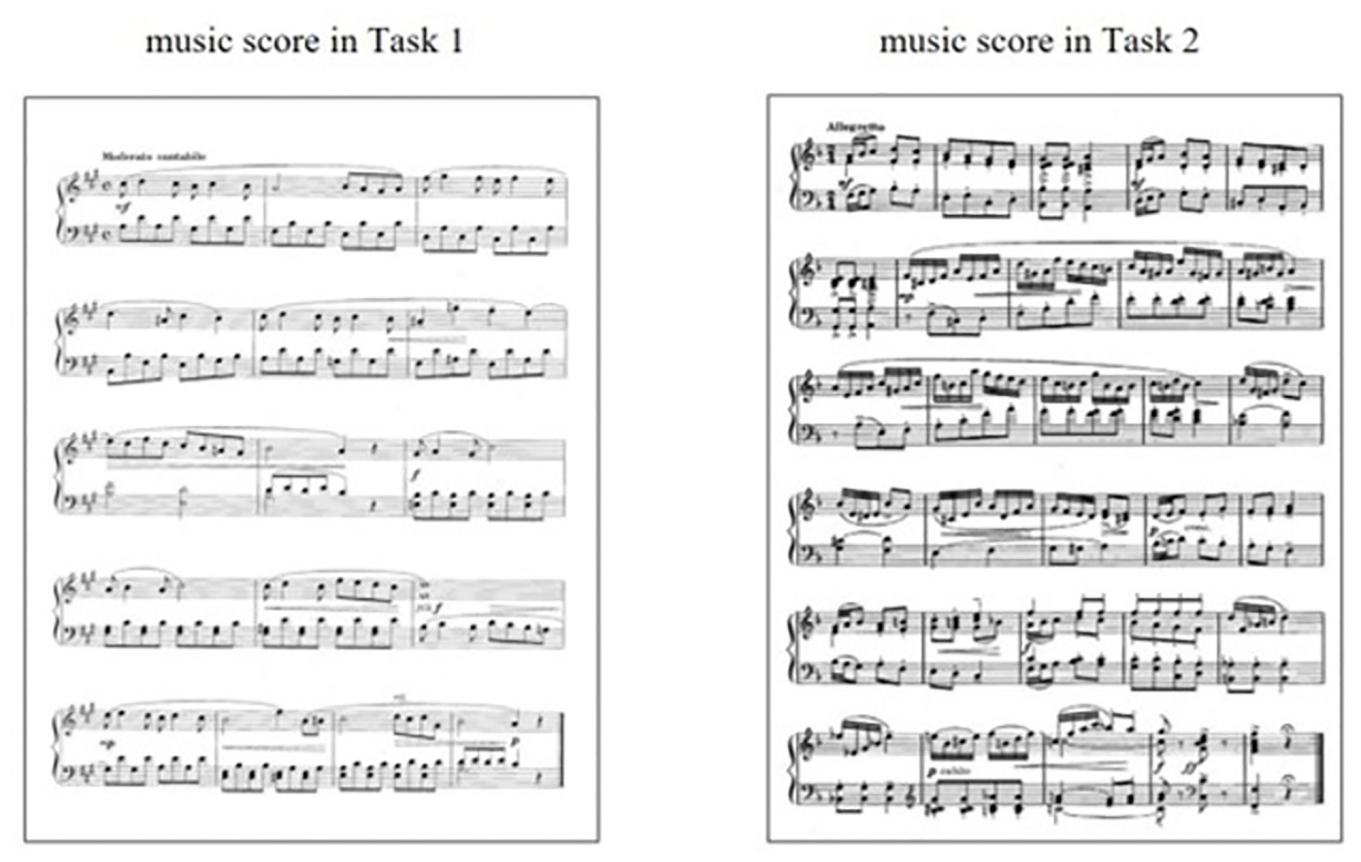
Music scores presented to the eye tracker.

From this workbook, we prepared two piano scores for the participants: grades 5 and 3 (Fig 1). The grade 5 music score (16 bars) was included as Task 1, and the grade 3 music score (30 bars) was included as Task 2. The tempo of the Task 1 score was moderate and that of the Task 2 score was allegretto. Participants played each score using both hands. We confirmed that none of the participants had previously seen the music scores after their performance.

### Eye-tracking apparatus

**Fig 2:**
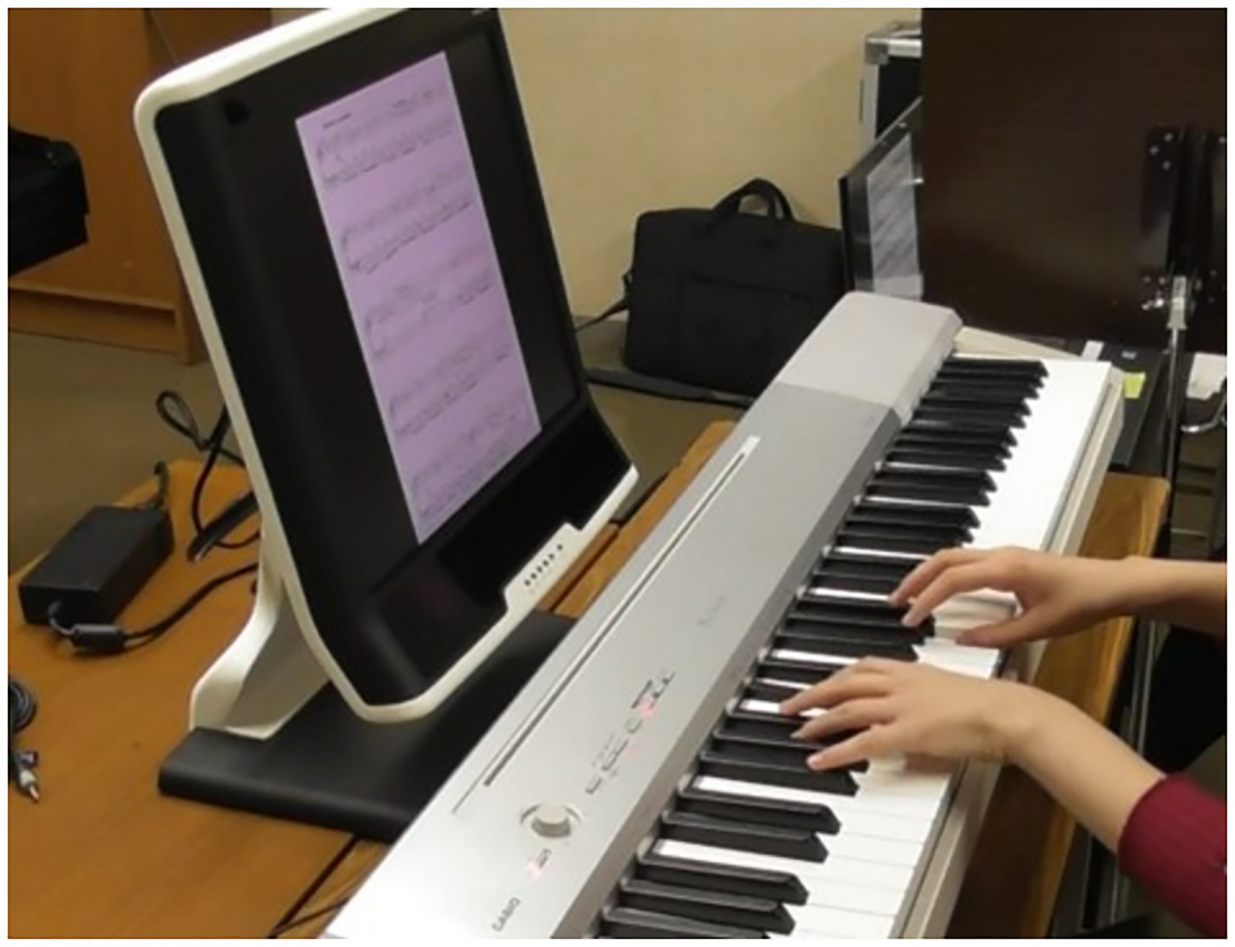
Participant sight-reading the music score presented on the eye-tracker.

We used the Tobii 17-inch display eye tracker Tobii T120 (Fig 2). Using this eye tracker, the gaze of both eyes can be measured without restraining the head. The sampling rate was set at 120 Hz. The resolution of the display was set to 1024 × 768 pixels. We placed a piano (CASIO privia PX-150 electronic piano with 88 keys) in front of the display, which was set to the correct height for playing. The computer operating the eye tracker was controlled by an experimenter and was set aside from the display, separated by a partition.

### Procedure

Calibration, measurement, and analysis were performed using Tobii Studio, an eye movement analysis software. The eye tracker was calibrated for each participant using a 5-point calibration, wherein the participant followed the location of a red dot shown on the screen with both eyes. If the calibration was incorrect, instructions appeared on the screen to try again. We followed the instructions and repeated the procedure when necessary.

After calibration, the participant viewed the Task 1 music score on the display screen for 60 s (sight-reading without piano performance). The experimenter next instructed the participant to set the tempo on the metronome and play the score on the piano. A fixation cross was shown on the center of the display screen for three seconds to fixate the participant’s gaze, followed by the display of the Task 1 music score. The participant played the piano while watching the music score (sight-reading with piano performance), and their gaze was measured. The piano performances were also recorded on a sound recorder to evaluate the performance.

After performing the easy music score task (Task 1) as described above, the same procedure was used to perform the difficult music score task (Task 2).

### Measurements

#### Computation of EHS using the eye tracker

We made video recordings of all subjects’ eye movements and piano sounds using Tobii Studio. EHS was measured by playing back videos recorded using Tobii Studio. When the subject played the first beat of each bar, the video was stopped, and the position of the subject’s gaze at that time was counted as the number of beats from the first beat of the bar. For each participant, the number of beats of eye-hand displacement for all 16 measures in the easy score and all 30 measures in the difficult score were examined, and the average value was calculated for each music score. In this study, EHS was defined as the number of beats of displacement between the eyes and hands.

#### Directly assessed EFs

*Shifting*. Shifting is the ability to switch tasks flexibly [27]. We used the Trail Making Test (TMT) to measure shifting. The TMT, one of the most common neuropsychological tests, provides information on visual search, scanning, processing speed, mental flexibility, and EF [28]. The TMT consists of two parts. The TMT-A consists of drawing a line connecting 25 numbers on a sheet of paper in sequence, while the TMT-B consists of connecting 13 numbers and letters in alternating order. The score for each of these tasks is expressed in terms of the time (in seconds) taken to complete the task. In the present study, the TMT-B was used as a measure of shifting, as it was mainly related to WM and set-shifting ability [29]. The smaller the score, the greater the shifting ability.

*Visual and verbal inhibitions*. We used a Stroop and a reverse-Stroop test as measures of visual and verbal inhibition [30, 31]. The test consisted of four tasks using a color name word stimulus that combined color and meaning, and five different color patches (red, blue, yellow, green, and black) printed on the screen. After practicing 10 questions for each task for 10 s, participants were given 60 s to answer 100 questions for each task, and the number of correct answers was obtained. Then, the Stroop interference rate (inhibition of verbal interference) and the reverse-Stroop interference rate (inhibition of visual interference) were calculated. The smaller the score, the greater the ability of inhibition.

*Visuospatial WM*. To assess visuospatial WM, we used the Kaufman’s hand movement task [32]. In this task, an assessor shows the participant three hand movements: tapping a desk with the fist, palm, and side of the hand. The assessor performs these movements in a specific order, which the participant is then asked to copy. The total scores can range from 0 to 23. The higher the score, the higher the WM.

*Auditory WM*. To assess auditory WM, we used the digit span backward task [33, 34]. In this task, the assessor states a series of numbers, and the participant repeats them back in the reverse order. It begins with two pairs of two-digit series presented one at a time. If the participant answers one correctly, they continue to two three-digit series, and so on, up to two eight-digit series. Total scores range from 0 to 16, based on the number of levels the participant completes successfully. The higher the score, the higher the WM.

#### Evaluation of performance

To determine the level of each participant’s performance, two pianists who were not participating in the experiment were asked to evaluate each participant’s recorded piano performance. The evaluators independently and blindly evaluated each performance from five perspectives: number of sound errors, beat and rhythm, tempo, quality of sound, and accent of sound. Each category was assigned 10 points, for a total of 50 points. The average of the scores from the two evaluators was calculated for each participant. Evaluators were given reward money.

To determine the reliability of the performance evaluation, a correlation coefficient between the scores of both evaluators was calculated. The Pearson correlation coefficients were *r* = .797, *p* < .001 for Task 1 and *r* = .910, *p* < .001 for Task 2. Because there was a significant correlation between both evaluators’ scores, their ratings were considered reliable.

### Data analysis

IBM SPSS Statistics and Amos Graphics Version 27 were used for structural equation modeling and statistical processing.

## Results

We investigated the extent to which the subcomponents of a pianist’s EF are involved in EHS, and to what extent EHS predicts performance in piano playing. Table 1 presents descriptive statistics for all participants and tasks.

**Table 1:** Descriptive statistics.

Measures	M	SD	Min	Max	Skewness	Kurtosis
Age	36.54	15.38	19	65	0.37	–1.35
Years of experience	33.33	15.45	14	62	0.40	–1.26
Shifting	53.06	7.76	35.00	60.23	–0.87	–0.41
Reverse-Stroop interference (visual)	0.19	0.08	0.02	0.36	–0.21	–0.28
Stroop interference (verbal)	0.10	0.12	–0.15	0.44	0.53	0.83
Visual working memory	15.79	3.06	7	22	–0.34	0.86
Auditory working memory	9.92	2.94	6	16	0.41	–0.49
EHS Task 1	1.49	0.73	0.40	3.40	1.06	0.50
EHS Task 2	1.10	0.86	0	3.00	1.09	0.06
Performance in Task 1	30.91	9.67	7.00	48.50	–0.24	0.22
Performance in Task 2	20.86	12.17	1.00	49.00	0.99	0.45

*Notes*. M = mean; SD = standard deviation; Min = minimum; Max = maximum; EHS = eye-hand span

The bivariate correlations of the variables are presented in Table 2. EHS was significantly positively correlated with auditory WM in both tasks 1 and 2 (*r* = .732, *p* < .001 in Task 1; *r* = .665, *p* < .001 in Task 2). Piano performance was significantly positively correlated with auditory WM (*r* = .639, *p* < .001 in Task 1; *r* = .662, *p* < .001 in Task 2) and EHS (*r* = .743, *p* < .001 in Task 1; *r* = .722, *p* < .001 in Task 2). There were no significant correlations between shift, two types of inhibition, or visual WM and EHS or performance. Age and years of experience were significantly correlated with visual and auditory WM, EHS, and performance in task 1.

**Table 2:** Correlations among all variables.

	**1**	**2**	**3**	**4**	**5**	**6**	**7**	**8**	**9**	**10**	**11**
**1. Age**											
**2. Years of experience**	**.993** **										
	**< .001**										
**3. Shifting**	**0.171**	**0.19**									
	**0.297**	**0.246**									
**4. Inhibition of visual interference**	**-0.136**	**-0.16**	**-.331** *								
	**0.41**	**0.33**	**0.04**								
**5. Inhibition of verbal interference**	**-.422** **	**-.421** **	**-.413** **	**.696** **							
	**0.008**	**0.008**	**0.009**	**< .001**							
**6. Auditory working memory**	**.321** *	**.323** *	**-0.277**	**0.287**	**0.034**						
	**0.046**	**0.045**	**0.088**	**0.077**	**0.836**						
**7. Visual working memory**	**-.373** *	-.366*	**-0.058**	**.346** *	**.397** *	**0.13**					
	**0.019**	**0.022**	**0.727**	**0.031**	**0.012**	**0.431**					
**8. EHS in Task 1**	**.480** **	**.481** **	**-0.081**	**.317** *	**-0.034**	**.732** **	**0.141**				
	**0.002**	**0.002**	**0.625**	**0.049**	**0.837**	**< .001**	**0.393**				
**9. EHS in Task 2**	**.358** *	**.367** *	**-0.029**	**0.255**	**-0.097**	.665**	**0.262**	**.846** **			
	**0.025**	**0.021**	**0.859**	**0.118**	**0.559**	**< .001**	**0.107**	**< .001**			
**10. Evaluation of performance in Task 1**	**.375** *	**.370** *	**-0.143**	**0.203**	**-0.164**	**.639** **	**0.069**	**.743** **	**.692** **		
	**0.019**	**0.02**	**0.385**	**0.215**	**0.318**	**< .001**	**0.676**	**< .001**	**< .001**		
**11. Evaluation of performance in Task 2**	**0.289**	**0.285**	**-0.294**	**0.248**	**0.017**	**.662** **	**0.168**	**.746** **	**.722** **	**.853** **	
	**0.075**	**0.078**	**0.069**	**0.129**	**0.917**	**< .001**	**0.306**	**< .001**	**< .001**	**< .001**	

The upper number in each square indicates the correlation coefficient and the lower number indicates the p-value.

* p < .05,

** p < .001

N = 39

### Relationships among each EF element, EHS, and performance

**Fig 3:**
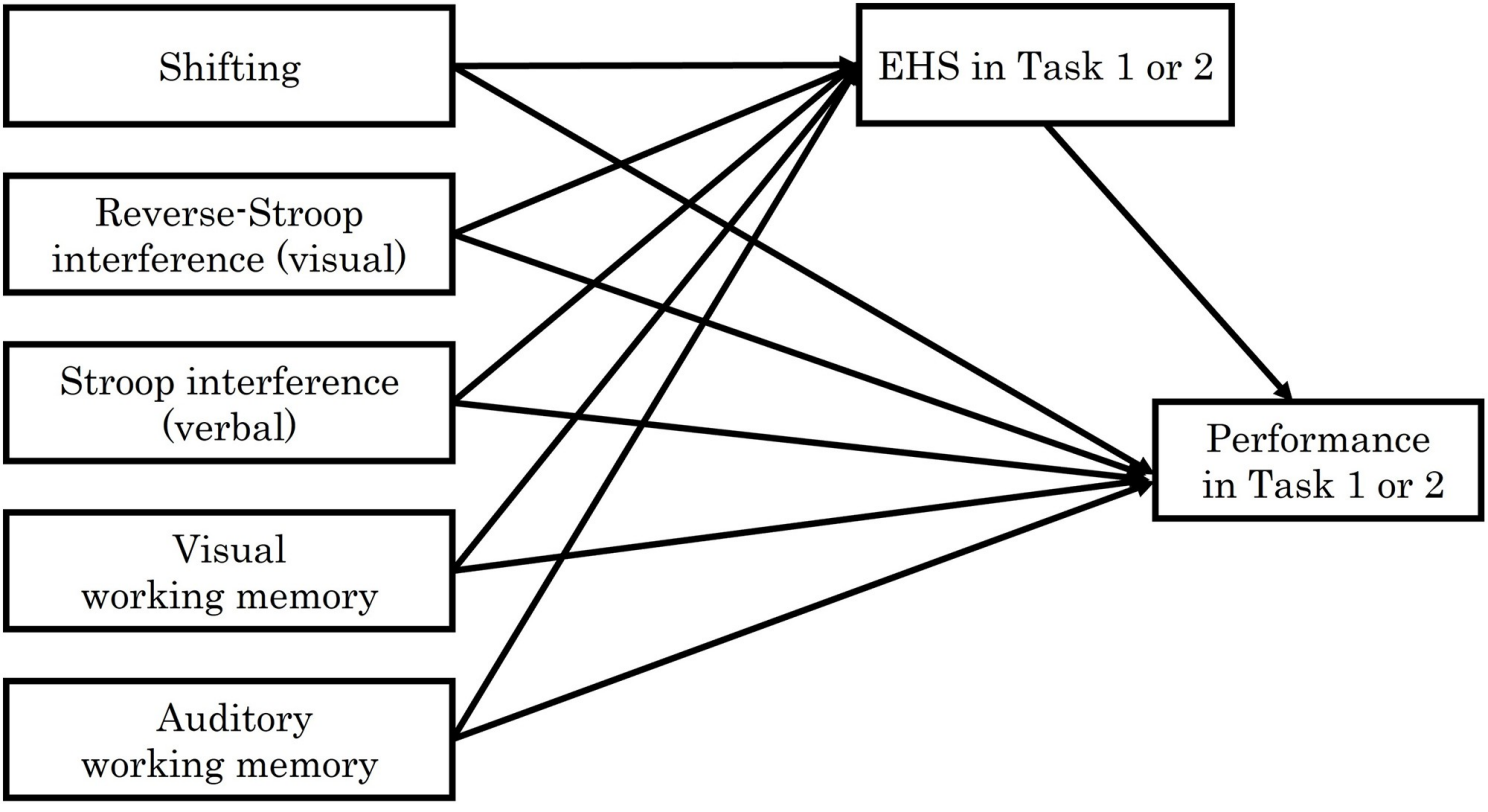
A model of relationships among each executive element, EHS, and piano performance.

IBM SPSS Amos Graphics version 27 was used to estimate the path model. Five variables of EF were set as predictors of EHS and performance. Error variables were also set for the endogenous variables of EHS and performance. The model is shown in Fig 3. Based on this model, we obtained the maximum likelihood estimates. We evaluated the fit of the models after trimming, and the following fit indices and criteria were used: *p* > .05, *CFI*, and *TLI* ≥ .95, *RMSEA* ≤ .06, and *SRMR* ≤ .08 [35].

**Fig 4:**
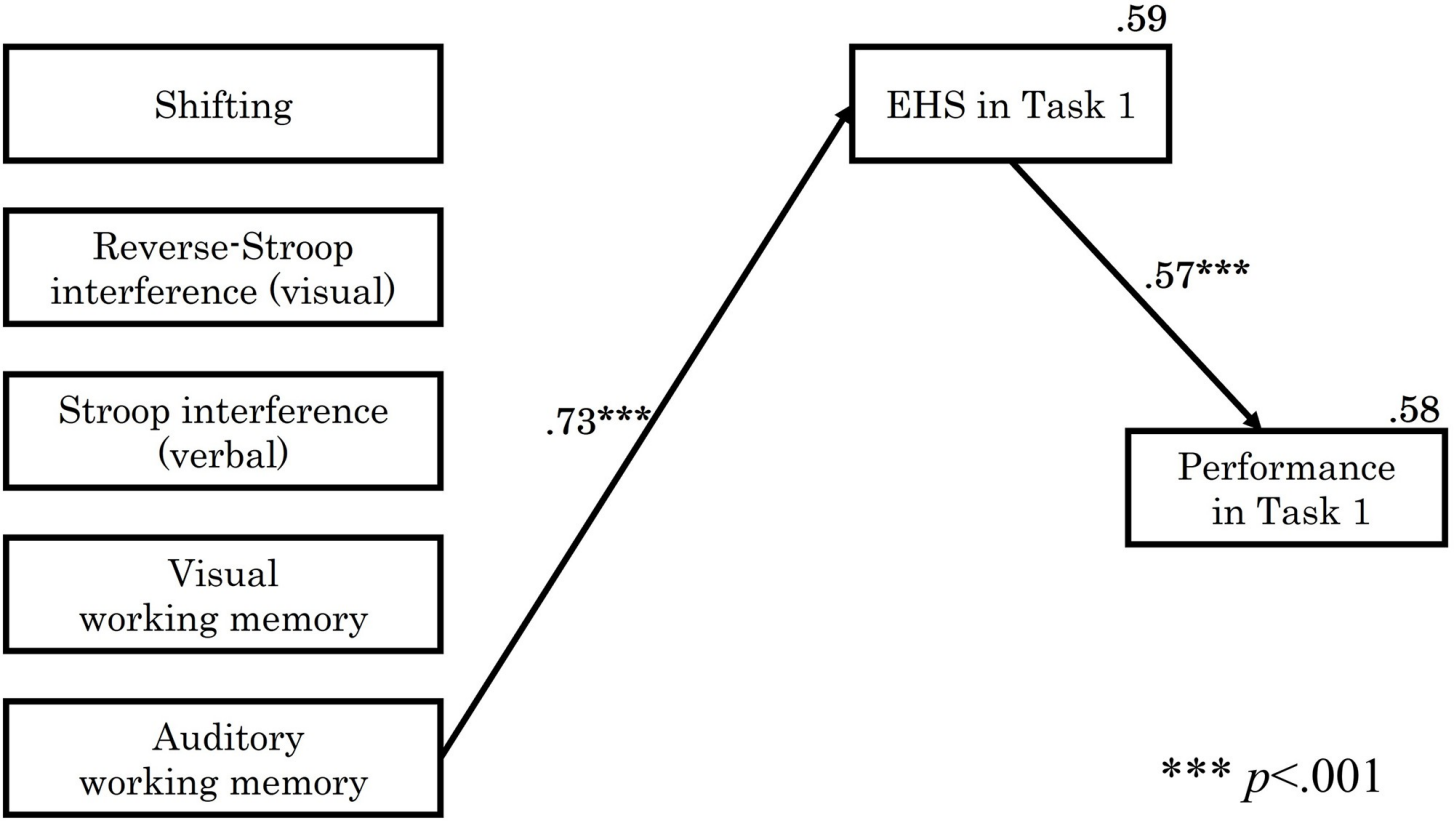
Results of path analysis in task 1. The figure shows only significant paths.

In the easy score (Task 1), the relationship among each EF element, EHS, and performance was examined by structural equation modeling using maximum likelihood estimation. The paths that were not significant at the 5% level of significance were removed, and the final model with the best fit is shown in Fig 4. The indices of goodness of fit were: *p* = .584, *CFI* = 1.000, *TLI* = 1.057, *RMSEA* < .001, and *SRMR* = .109. The *SRMR* was slightly higher, but all the other indices met the criteria.

The results of the analysis of Task 1 showed that of the EF elements, only auditory WM predicted EHS (*β* = .73, *p* < .001). This effect explains 59% of the EHS (*R*^*2*^ = .59). No direct path from EF to performance was found. However, a significant path from EHS was found (*β* = .57, *p* < .001), which explained 58% of performance (*R*^*2*^ = .58). This indicates that higher auditory WM ability promotes higher EHS and that increased EHS improves piano performance.

**Fig 5:**
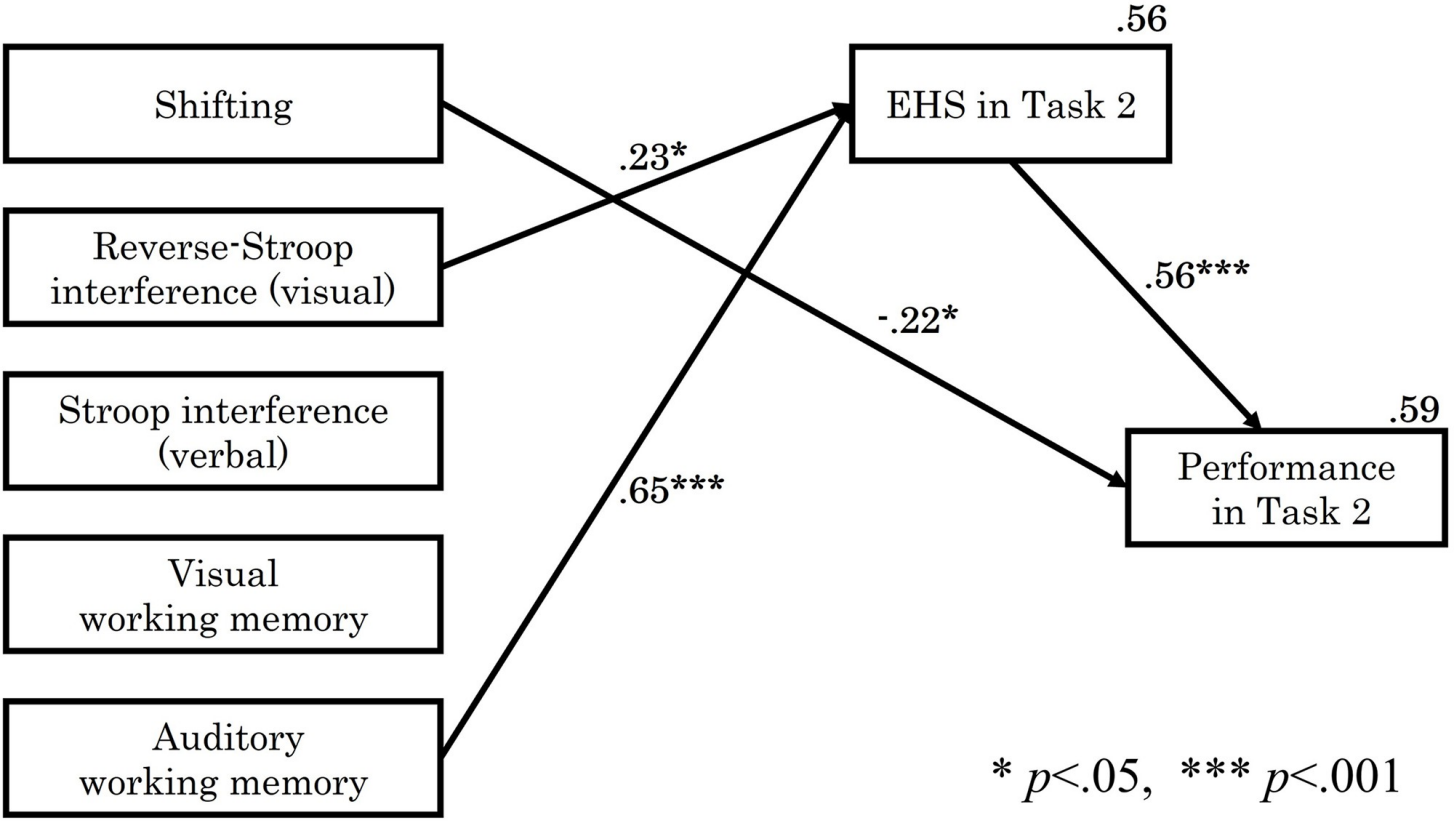
Results of path analysis in task 2. The figure shows only significant paths. For Shifting or Stroop, the lower values indicate a higher ability to switch or inhibit.

In the difficult score (Task 2), the relationship among each EF element, EHS, and performance was examined by structural equation modeling using maximum likelihood estimation. The paths that were not significant at the 5% level of significance were removed, and the final model with the best fit is shown in Fig 5. The indices of goodness of fit were: *p* = .584, *CFI* = 1.000, *TLI* = 1.059, *RMSEA* < .001, and *SRMR* = .112. The *SRMR* was slightly higher, but all the other indices met the criteria.

The results of Task 2 analysis showed that among the EF elements, auditory WM (*β* = .65, *p* < .001) and the reverse Stroop interference rate (inhibition of visual interference) (*β* = .23, *p* = .029) predicted EHS. This effect accounted for 56% of EHS (*R*^*2*^ = .56). In other words, the higher the auditory WM ability, and the weaker the visual inhibition, the longer the EHS. The effect of auditory WM was particularly significant. There was a direct negative path from shifting to performance in Task 2 (*β* = -.22, *p* = .040), although there was no direct path from EFs to performance in Task 1. The results of Task 2 indicate that higher shifting abilities lead to better performance since lower shifting values indicate greater ability. In addition, the performance received a significant pass from EHS (*β* = .56, *p* < .001), with these two passes explaining 59% of performance (*R*^*2*^ = .59). This indicates that EF improved EHS and that increased EHS also improved piano performance. It was also found that shifting ability was directly involved in performance during sight-reading of complex scores.

### Effect of complexity in music scores on EHS

A t-test was conducted to examine the differences in EHS depending on the difficulty of the music score. The results showed that EHS was significantly greater for the easy score (Task 1) than for the more difficult score (Task 2), *t* (38) = 5.242, *p*< .001, *d* = .457. The effect size was medium.

### Do years of experience predict EHS, performance, or EFs?

We examined whether years of experience in playing the piano predicts EHS, piano performance, and EFs. Using these variables as dependent variables and years of experience as independent variables, we conducted a single regression analysis. Years of experience significantly predicted EHS in both Task 1 (*R* =. 481, *R*^*2*^ = .231, *p* = .002) and Task 2 (*R* = .367, *R*^*2*^ = .135, *p* = .021), and performance in Task 1 (*R* = .370, *R*^*2*^ = .137, *p* = .020). For effects on EFs, years of experience significantly predicted only auditory WM (*R* = .323, *R*^*2*^ = .104, *p* = .045) and visual WM (*R* = .366, *R*^*2*^ = .134, *p* = .022).

## Discussion

The present study is the first to clarify the involvement of elements of EF in EHS and piano performance during sight-reading. The purpose of this study was to determine which subcomponents of EF are involved (and to what extent) in EHS and piano performance during sight-reading. Two conditions were particularly important for this study: the participants had to be able to perform sight-reading immediately after seeing the music score for the first time, and the eye-hand span had to be measured accurately. Therefore, in this study, the participants were professional pianists and aspiring professional students with 14–62 years of piano experience. EHS was measured using an eye tracker, using beats as an index. Since EHS is affected by the difficulty of a music score [24], sight-reading was performed with two scores of different difficulty levels. Sight-reading piano performance was evaluated as the average of two professional pianists’ blind, independent ratings.

The results were clear: auditory WM predicted EHS (accounting of approximately 60% of the EHS score) in both easy and difficult scores (Task 1 and 2). None of the subcomponents of EF were directly involved in piano performance of the easy score (Task 1), whereas the shifting ability predicted the performance directly during the sight-reading a difficult score (Task 2). Furthermore, in both scores, EHS predicted piano performance, accounting for approximately 60% of the total.

### Prediction of EHS by auditory WM

EHS has been regarded as a decisive indicator of performers’ competence in sight-reading. However, integrated perspectives regarding the relationship between EHS and sight-reading variables have not been discussed in depth. Among them, the relationship between the EHS and WM has been hypothesized but not reported in practice. For example, Rosemann, Altenmuller, and Fahle [2] measured the EHS of nine pianists with an eye-tracker, and examined the relationship between EHS and cognitive function, but did not find a significant relationship with WM. However, they cited the small sample size and head fixation of the eye-tracker as limitations of the study. Lim et al. [21] also measured EHS with a glasses-type eye-tracker in 31 piano majors with significant piano experience, but did not find a relationship with cognitive function. In addition, Gagnon and Nicoladis [18] compared the visual, verbal, and motor WM of musicians and non-musicians, and found no significant differences between the two groups for any WM type. They attributed this to the fact that they could not identify the abilities of non-musicians. They also did not measure EHS in their study. As hypothesized in these previous studies, in the current study, we hypothesized that WM would predict EHS. As we predicted, out of the subcomponents of EF, auditory WM was the component that predicted EHS. Auditory WM was strongly involved, but visual WM was not.

The present study found that greater auditory WM was associated with greater EHS, and greater EHS was associated with better performance. This suggests that the input of notes from the eyes by looking at the music score becomes sound in the brain and activates the auditory WM, which is then transmitted to finger movement, resulting in piano performance.

WM is a cognitive system that temporarily stores, rehearses, updates, manipulates, and integrates internally stored information within a limited storage capacity [36]. In Baddeley’s model [37], it consists of the phonological loop (maintaining and manipulating linguistic information), the visuospatial sketchpad (maintaining and manipulating visuospatial information), and the central executive. EHS is thought to be processed with the involvement of the phonological loop.

Furthermore, the involvement of EFs was found to be more important for EHS during sight-reading a difficult score than on an easy score. The reverse Stroop interference rate (inhibition of visual interference) has also been shown to predict EHS, although not as significantly as auditory WM. In other words, the EHS increased when interference was not inhibited in the presence of visual interference. Additionally, shifting ability was directly related to performance. These findings suggest that sight-reading with higher difficulty scores requires more advanced EFs.

### EF and piano performance during sight-reading

Our hypothesis that each participant’s EFs were directly related to piano performance was partially supported. We found that higher shifting ability was associated with higher performance during the sight-reading of a difficult score. On an easier score, however, EFs were not directly involved in the piano performance.

Kopiez and Lee [15, 16] measured a variety of cognitive functions in piano students and graduates, and examined their relationship with sight-reading performance. Their definition of sight-reading was similar to that in the current study, but their performance assessment was based on a software-based measure of errors with the music score. The results showed that WM was not involved in piano performance. Meinz and Hambrick [17] also examined whether deliberate practice and WM predicted piano performance in subjects with piano-playing experience, using a similar definition of sight-reading and a similar method of assessing piano performance. However, their results showed that deliberate practice and WM were predictive of piano performance, but the explanatory variable for deliberate practice was large (*R*^*2*^ = .451), while WM was small (*R*^*2*^ = .074). Our results also showed that auditory WM did not directly predict piano performance, but through EHS.

### Prediction of sight-reading piano performance by EHS

#### EHSによる視唱能力の予測

In Tasks 1 and 2, the EHS strongly affected performance and had a high coefficient of determination.

Previous research has shown that skilled players display higher EHS [8, 22, 25, 26]. The results of the present study are consistent with previous findings. Furthermore, the present study showed not only a relationship between EHS size and pianist proficiency but also that a larger EHS predicted better sight-reading piano performance. Therefore, it was important to clarify these findings.

### The complexity of music score and EHS

Based on previous studies [21], we predicted that the EHS would be longer for uncomplicated and easy scores than for complex and difficult scores. This study supported this hypothesis. In addition, as we hypothesized, the score complexity also affected the relationship between EF and EHS. For less complex scores, the EHS was predicted by auditory WM alone. However, for more complex scores, the EHS involved not only auditory WM but also visual interference inhibition. The weaker the inhibition of visual interference, the larger the EHS. We do not currently know the reason for this phenomenon. In both scores, auditory WM is definitely an important factor in lengthening the EHS.

Furthermore, for a difficult score, the higher the shifting ability, the better the piano performance. The results suggest that piano performances with difficult scores require the ability to switch quickly between eye movements and finger, hand, and foot movements.

### Multisensory integration based on visual, auditory and motor interactions during sight-reading

To maintain high accuracy levels in their performance, pianists use various sensors in the body (eyes, ears, skin, and muscles). It has been reported that pianists possess such specialized abilities, which are acquired through training from an early age [38]. For example, it is known that just listening to a piece of music in pianists activates the motor cortex of the brain, even without moving the fingers [39, 40]. Pianists also move their fingers naturally when listening to or imagining a song [5]. They have special brain and body functions that automatically convert sounds they hear to their fingers and hands.

Furthermore, in sight-reading, where a pianist plays a music score for the first time, the pianist is required to play at the same time as seeing the score. Within a split second, they can determine proper fingering. This sequence of processes occurs in the EHS. In the EHS, they gaze at the note ahead of the one they are fingering and then perceive the surrounding 2–4 beats of notes with peripheral vision [25]. From the notes read, the fingerings were selected as appropriate for playing these notes by recalling memories. If a pianist strikes a note with an inappropriate finger, it will be difficult to play the next note. Therefore, the order in which notes are played with the fingers is determined by considering the preceding and succeeding notes. The EHS process is momentary and continues until the piece is completed. This would not have been possible without WM.

Our results indicate that auditory WM, not visual WM, is critical for EHS. This suggests that pianists look at scores and store notes as auditory rather than visual information. Furthermore, we assume that sound replaces the reference fingering memory.

The difficult score also showed that the weaker the ability to inhibit visual interference, the larger the EHS. It is conceivable that visual inhibition ability is not strong enough to be related, but it is unclear why this is the case.

Furthermore, for the difficult score, the higher the shifting ability, the higher the evaluation of actual performance. Shifting ability may be necessary for multisensory visual, auditory, and motor interaction integration.

In summary, although previous studies have shown that excellent pianists have superior cognitive processing speed, possibly due to a longer eye-hand span [41, 42], the present study suggests that auditory WM may be particularly significant.

### Limitations and future research directions

The present study has several limitations and future challenges. The first was the wide range of ages and years of piano experience of the participants in this study. Different piano proficiency leads to EHS differences. This study found that years of experience affected EHS, piano performance, and auditory and visual WM. However, as EF changes with age [43, 44], more detailed studies are needed to address this.

Second, it is known that EF develops at an early age, and piano lessons generally begin at earlier ages. The development of EF, EHS, and their interrelationships are unknown.

Third, the present study used two music scores with different difficulty levels and showed that the EHS differed in both scores. As the modality of a score affects the efficiency of input and visuomotor coordination in an advanced pianist’s sight-reading [45], it may also be necessary to clarify the difference between minor and major keys.

## Conclusion

This study hypothesized that WM is involved in EHS observed in skilled piano players during sight-reading and tested whether EFs, including WM, are involved in EHS and whether EHS and its subcomponents predict the piano performance level. To this end, piano players capable of sight-reading were used as subjects, and EHS was measured using an eye tracker with two music scores at different difficulty levels. Structural equation modelling showed that auditory WM predicted EHS and that EHS predicted piano performance. This suggests that visual input from the eyes becomes sound in the brain, activating auditory WM, which is transmitted to finger movements and piano performances. The EHS of the difficult scores was significantly shorter than that of the easy scores. Furthermore, shifting ability predicted directly piano performance during sight-reading a difficult score.

## Supporting information

S1 Data (XLSX)
